# Vertical Jumping Performance: Recording the Effects of Proprioceptive Neuromuscular Facilitation Stretching at Different Plantar Flexor Lengths

**DOI:** 10.7759/cureus.43346

**Published:** 2023-08-11

**Authors:** Theodoros M Kannas, Georgios Stefanis, Apostolis Kousinas, Georgios Chalatzoglidis, Fotini Arabatzi

**Affiliations:** 1 Laboratory of Neuromechanics, Department of Physical Education and Sport Science, Aristotle University of Thessaloniki, Serres, Thessaloniki, GRC; 2 Laboratory of Neuromechanics, Department of Physical Education and Sport Science, Aristotle University of Thessaloniki, Serres, GRC

**Keywords:** jumping height, proprioceptive neuromuscular facilitation (pnf), ground reaction forces, ankle joint, tricep surae

## Abstract

Introduction: Flexibility seems to be an essential part of both the training and rehabilitation processes. Several stretching techniques have been used to improve the range of motion (ROM) of the joints with the proprioceptive neuromuscular facilitation (PNF) method being the most effective one. Although plantar flexors are ideal to compare the acute effects of synergistic muscle groups on performance, it is not clear whether the PNF stretch at different muscle lengths could result in different alterations.

Material and methods: Sixteen male students randomly performed 2 levels of stretching (PNF with bended knees, or PNFshort, and with extended knee, or PNFlong) and 3 types of jumps, separated by 48 hours (7 sessions in total). Jumping parameters were recorded by a force plate, and the final jumping height (H) and ground reaction forces (Fz) were analyzed. Furthermore, the ROM of the ankle joint was recorded before, right after, and 15 minutes after the stretches.

Results: The ankle joint’s ROM joint was increased after both interventions. No significant changes were found in the jumping height of all jumps. The Fz, during the squat jump (SJ) and countermovement jump (CMJ), were increased after PNFshort. Similarly, a significant increase was found in Fz in drop jumps (DJ) right after the PNFshort.

Conclusion: Our findings demonstrated that PNF stretches of different lengths could potentially alter the stretch-shortening cycle’s performance, possibly leading to a non-optimal muscle-tendon interaction.

## Introduction

Flexibility is an important factor in both sports performance and the rehabilitation process [1,2]. Several stretching techniques are effective in increasing range of motion (ROM), with static stretching being the most commonly used [3]. Concerning athletic performance, a previous report [4] showed that static stretching might harm an athlete’s explosive performance, such as jumping due to mechanical properties and neural activation alterations [5]. However, a recent study [6] showed that no type of stretching (neither static nor dynamic) affected physical performance when included in a complete specific warm-up routine, or even had a post-stretching potentiation effect [7]. Despite the wide use of static stretching, proprioceptive neuromuscular facilitation (PNF) stretches are considered more effective for improving ROM [8,9,10].

The effectiveness of PNF stretches has been previously established for different muscle groups [9, 10]. It was reported that the dorsiflexion angle was improved by the use of PNF [10], indicating that the superiority of PNF, compared with static stretching, relies on the combination of static stretching and isometric contraction. Both static stretch and isometric contraction reduce muscle and tendon stiffness, respectively, leading to a better final result than when these techniques are used separately. Furthermore, most previous studies included stretches targeting specific muscles [6] or one muscle group [11]. However, there are limited data about the acute effects of PNF stretching on synergist muscles, such as the plantar flexors, especially just prior to explosive activities.

A 6-week PNF intervention program led to an increased ankle ROM, without any differences in passive resistive torque and the stiffness of the Achilles tendon, possibly due to improved stretch tolerance [12]. Similarly, the contract-relax method resulted in greater improvements in ankle ROM, compared with static stretching or isometric contraction used separately, using the previous protocol [13]. They proposed that static stretch and isometric contraction reduces muscle and tendon stiffness, respectively, leading to a greater ROM [13]. The above results highlight the properties of the musculotendinous system as well as stretch tolerance as the most important factors for the observed changes through PNF stretching.

Although a variety of studies focus on the plantar flexors' adaptation after PNF stretches [12,13], there are no available data about the acute effects of plantar flexors' PNF stretch applied at different knee joint angles. Therefore, the purpose of the present study was to investigate whether a single session of modified PNF stretching of the triceps surae muscles, at different muscle lengths, affects jumping performance.

## Materials and methods

Participants

G*Power software (version 3.1.9.7; Heinrich-Heine-Universität Düsseldorf, Düsseldorf, Germany) was used for the statistical power analysis. The pοwer analysis indicated that a total sample size of 16 participants was required to achieve an actual power of .84. The parameters used for sample size estimation were; effect size: .35, alpha level: .05, power (1-β): .80, and correlation among repeated measures: .50 [14]. In the F-test, there were two random variables involved: the variability between groups (numerator) and the variability within groups (denominator). The F-test was conducted with 2 degrees of freedom in the numerator (PNF has 2 levels, short and long); so, there were 2 groups to compare, and 28 degrees of freedom in the denominator (the total number of observations, 2 × 3, minus the total number of groups, 2). The critical F-value at a significance level of p < .05 was 3.34. The alternative hypothesis was that there was a significant interaction effect between length and time. This hypothesis implies that at least one combination of length and time levels results in significantly different mean values, suggesting an effect. The noncentrality parameter (λ) representing the effect size was calculated to be 11.76.

Male students in the age group of 17-21 years with no systematic training in a specific sport for the last 2 years, who were in an active state (10-12 hours/week) with no systematic strength or plyometric training in the last 6 months, and were willing to participate, were included in the study. In contrast, smokers and students using food supplements such as protein or creatine, or having a lower limb injury in the last 6 months were excluded from the study. Finally, 16 male students (age: 18.4 ± 1.8 years, height: 179.5 ± 3.2 cm, weight: 82.7 ± 3.5 kg) of the Department of Physical Education and Sports Sciences voluntarily participated, in this study. The experimental approval was obtained from Local Ethics Committee (ERC-008/2020). All the participants did not involve in any particular strength and/or plyometric training program over the last 6 months.

Experimental procedure

**Figure 1:**
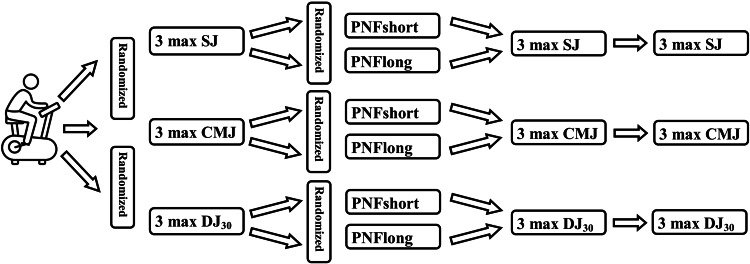
Experimental procedure SJ = squat jump, CMJ = countermovement jump, DJ_30 _= drop jump from 30 cm, PNFlong = proprioceptive neuromuscular facilitation with extended knees, PNFshort = proprioceptive neuromuscular facilitation with bended knees at 90°

Participants were asked to visit the laboratory on 7 separate days with 48 hours of rest between them. During the first visit, the participants familiarized themselves with the testing set-up, which included 3 maximal squat jumps (SJ; from a semi-squatting position the subject performed a maximum jump), countermovement jumps (CMJ; from a standing position the subject performed a semi-squat at 90°, and a maximal vertical jump), and drop jumps from 30 cm (DJ_30_; the subject was in a standing position on a raised platform at 30 cm), dropped from the platform and performed maximal vertical jump as fast as they could. All jumps were performed on a three-dimensional platform (Kistler Type 9281C, Kistler Instruments, Winterthur, Switzerland, sampling rate: 1000 Hz) while ground reaction forces (Fz) were recorded and compared (in Newtons). The participants randomly (Random Generator, free software) performed the PNF intervention with the knee extended (PNFlong) or with the knee bent at 90° (PNFshort). A twin-axis electronic goniometer, connected to BIOPAC MP100 unit (Biopac Systems Inc, Goleta, CA), was used to record ankle joint angles before and after the PNF interventions. All test sessions started with a 6-minute warm-up routine on a static bicycle. Following the warm-up, the baseline jumping test (depending on the day) was performed (TB) and repeated after the stretching intervention (T0). They rested for 15 minutes (T15) and then repeated the jumps (Figure 1). The best jump based on maximum height was used for further analysis.

Modified PNF stretches

The PNF condition involved stretching the tricep surae of both legs using the Contract Relax Antagonist Contract technique [12]. A 15-second static stretch of the muscles (either with the knee bent at 90° or fully extended knee) was followed by a maximal isometric contraction of the plantar flexor muscles for 6 seconds in the stretched position with the assistance of the researcher. After this isometric contraction, the subjects were asked to dynamically contract their dorsiflexor muscle (tibialis anterior) for another 15 seconds. During the stretching intervention, 5 sets of the aforementioned (Contract Relax Antagonist Contract) were repeated, with a 20 seconds rest between each set. The PNF stretch procedure has been previously described in a study [12].

Statistical analysis

The statistical analysis was performed in SPSS Statistics version 25.0 (IBM Corp., Armonk, NY). A two-way repeated measures ANOVA and pairwise comparisons were conducted between i) PNFshort and PNFlong, ii) TB, T0, T15, and iii) for interaction length x time, using Bonferroni confidence interval adjustment. Statistical significance was examined at p < .05. The Bonferroni adjusted significance level for each comparison .05/3 = .017. This means that for each individual comparison, we compared the p-value to .0167 instead of the original .05.

## Results

Range of motion

PNFshort (Mean = 37.454 ± 0.350) was greater [F (1,28) = 218.560, p < .001] from PNFlong (Mean = 30.132 ± 0.350) at three time points: TB, T0, and 15 (Table 1). The main effect of time was significant for both PNFshort and PNFlong [F (2,56) = 57.462, p< .001]. Post-hoc tests with Bonferroni correction showed significant differences in ROM between TB and T0 (p < .001), TB and T15 (p < .001), and T0 and T15 (p < .001) for both PNFshort and PNFlong.

**Table 1:** ROM (°) before and after the PNF interventions, $ indicates a significant difference (p < .05) between PNFshort and PNFlong at each time point, * indicates significant change (p < .05) compared to the previous time point ROM: range of motion, PNFlong: proprioceptive neuromuscular facilitation with extended knees, PNFshort: proprioceptive neuromuscular facilitation with bended knees at 90°, TB: baseline time point, T0: immediately after the stretch time point, T15: 15 minutes after the stretch time point

			Mean ± SD	95% CI	
				Lower bound	Upper bound
ROM (N=16)	PNFshort	TB	36.5° ± 0.3°$	35.8°	37.3°
		T0	38.4° ± 0.4°$*	37.6°	39.3°
		T15	37.3⁰ ± 0.3°$*	36.6°	38.0°
	PNFlong	TB	29.3° ± 0.3°	28.5°	30.0°
		T0	30.9° ± 0.4°*	30.1°	31.8°
		T15	30.1° ± 0.3°*	29.3°	30.7°

Squat jump

**Figure 2:**
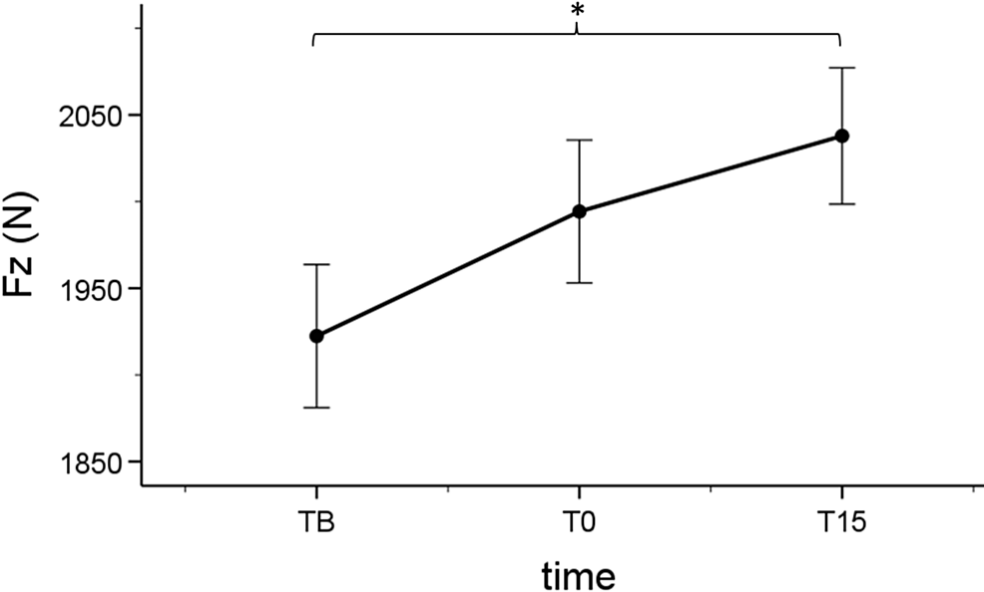
Fz values during SJ at the three time points, after the PNFshort condition, * indicates significant change (p < .05) compared to the previous time point Fz: ground reaction forces, SJ: squat jump, PNFshort: proprioceptive neuromuscular facilitation with bended knees at 90°, TB: baseline timepoint, T0: immediately after the stretch time point, T15: 15 minutes after the stretch time point

There was no effect of length [F (1,28) = 1.873, p = .182), time [F (2,56) = 1.660, p = .199], and interaction between length and time [F (2,56) = .559, p = .575] for jumping height (Figure 2). A significant interaction length x time was detected [F (2,56) = 3.348, p = .042] indicating that Fz at PNFshort T15 (Mean = 2037.867 ± 75.776 N) was greater than Fz at PNFshort TB [Mean = +115.467 ± 36.742 N (22.174 - 208.759), p = .012]. Fz at PNFlong was not different across time points in SJ. The main effect of time was close to significance [F (2,56) = 3.163, p = .050].

Countermovement jump

**Figure 3:**
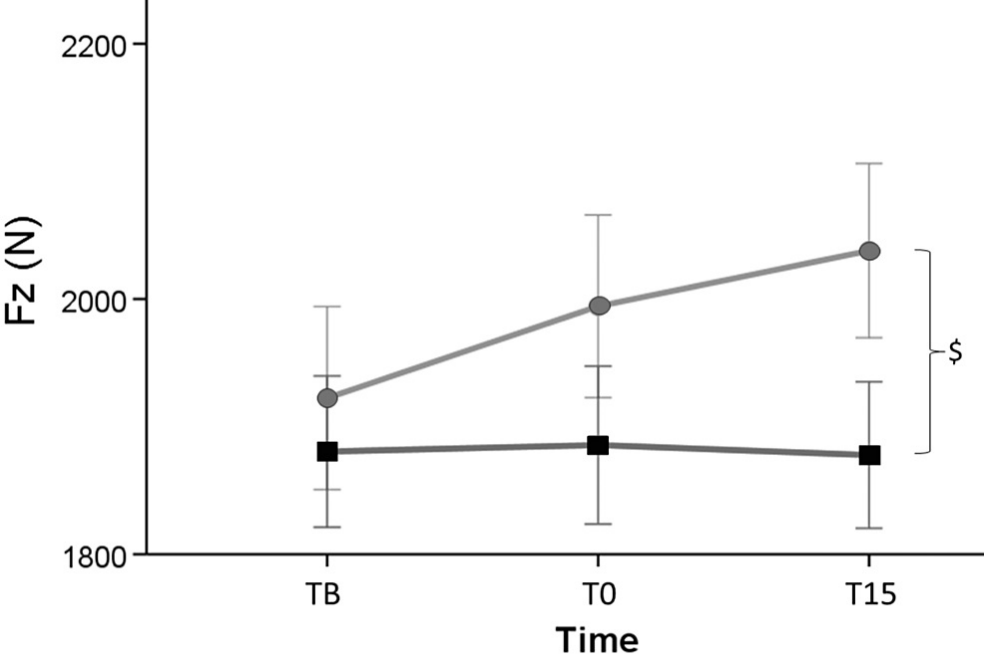
Fz at PNFlong (squares), Fz at PNFshort (circles) in CMJ at three time points, $ indicates a significant difference (p < .05) between PNFshort and PNFlong at each time point Fz: ground reaction forces, PNFlong: proprioceptive neuromuscular facilitation with extended knees, PNFshort: proprioceptive neuromuscular facilitation with bended knees at 90°, CMJ: countermovement jump, TB: baseline time point, T0: immediately after the stretch time point, T15: 15 minutes after the stretch time point

Height at PNFlong (Mean = 0.347 ± 0.012 m) was not significantly different [F (1,28) = 2.949, p = .097] from height at PNFshort (Mean = 0.319 ± 0.012 m) (Figure 3). No effect of time [F (2,56) = 1.205, p = .307] and no interaction between length and time [F (2,56) = 2.215, p = .119] were observed. Fz at PNFlong (Mean = 1900.889 ± 80.931 N) didn’t differ [F (1,28) = 3.627, p = .067] to Fz at PNFshort (Mean = 2118.867 ± 80.940 N) at time points: TB and T0 while Fz at PNFshort T15 found to be greater than Fz at PNFlong T15 [Mean= +274.333 ± 128.420 N (11.278 - 537.389), p = .042]. The main effect of time was significant [F (2,56) = 4.334, p = .018], and there was no interaction between length and time [F (2,56) = 2.316, p = .108].

Drop jump

**Figure 4:**
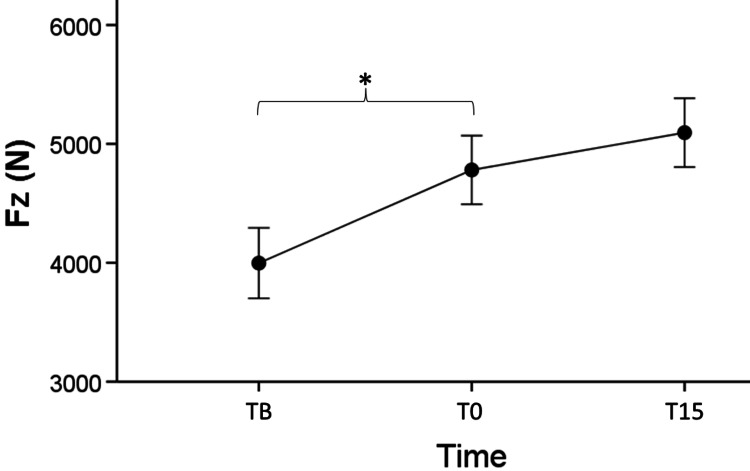
Fz values after PNFshort in drop jump from 30 cm at three time points, * indicates significant change (p < .05) compared to the previous time point Fz: ground reaction forces, PNFshort: proprioceptive neuromuscular facilitation with bended knees at 90°, TB: baseline time point, T0: immediately after the stretch time point, T15: 15 minutes after the stretch time point

There was no effect of length [F (1,28) = 3.164, p = .086], time [F (2,56) = 3.081, p = .054], and interaction between length and time [F (2,56) = 0.249, p = .780] (Figure 4). There was no effect of time [F (2,56) = .644, p = .529] and interaction between length and time [F (2,56) = 1 .974, p = .148]. Fz at PNFlong (Mean = 4582.400 ± 336.012 N) and Fz at PNFshort (Mean = 4624.133 ± 336.030 N) were similar [F (1,28) = .008, p = .931]. There was significant interaction between time and length [F (2,56) = 6.496, p = .003]. Time was also a significant factor [F (2,56) = 5.936, p = .005]. Post hoc analysis showed that only Fz at PNFshort T0 (Mean = 4780.533 ± 338.527 N) was greater than Fz at PNFshort TB (Mean = +783.267 ± 271.740 N [95% CI 93.287 - 1473.2245], p = .022). Although Fz at PNFshort T0 was lesser than Fz at PNFshort T15, that was not significant (p = .240). Fz at PNFlong was not different across time points in DJ.

## Discussion

The primary aim of the present study was to evaluate the effects of PNF stretching, with the knee either extended or flexed, on vertical jump performance. Both PNF interventions resulted in a significant improvement in the ankle’s ROM immediately after the stretching intervention, with the ROM returning to baseline values 15 minutes after stretching. Regarding vertical jumping, significant increases were observed in the Fz following PNFshort, despite no significant changes in vertical jumping height observed with either of the PNF interventions.

Range of motion

Consistent with previous reporting using similar PNF stretching techniques, both PNFshort and PNFlong interventions led to significant increases in the ankle’s ROM [10]. These findings align with other studies demonstrating that an improved ROM can be induced by a single bout of PNF stretching [10] or an intervention period [13]. An important factor contributing to these changes could be the ankle angle during the stretch. In our study, similar to a previous one [10], participants performed ankle stretches with a newly achieved ROM in every repetition. In contrast, previous studies [15] used a constant angle during the whole session, possibly leading to sub-maximum achievable dorsiflexion. Consequently, the stretch stimulus was higher in every repetition due to the new dorsiflexion angle. Moreover, previous research [12] reported improvements in the ankle’s ROM due to modified PNF stretching, highlighting the initial angle of ROM as an important factor for these changes. The different initial ROM among our male student participants might also explain the greater ROM increases compared to other studies with different participant groups.

Squat jump

Our findings indicated that both PNFshort and PNFlong did not alter the jumping heights during SJ, suggesting similar effects on jumping performance. These results are consistent with previous studies that also found no significant effects of PNF stretching on jumping height [6,16]. It is possible that the force capacity of the plantar flexors might not have caused any significant reductions in SJ jumping height [17,18]. Moreover, the sub-maximal level of force produced during PNFshort, due to the bended knee position, may have limited the possible negative effects on jump performance. Additionally, the PNFlong intervention, involving maximal isometric force and a greater stretch, led to a stable performance [19]. Furthermore, the non-optimal interaction between force production and propulsion time during PNFshort might explain the non-significant increase in Fz during SJ. On the other hand, PNFlong might have resulted in altered Achilles tendon properties, but the 15-minute rest period was sufficient for the tendon to restore its properties, hence no significant effects on jumping performance were observed.

Countermovement jump

Neither parameter of CMJ performance was altered following either stretching stimulus (PNFshort and PNFlong). The aforementioned findings agree with a previous finding showing that PNF stretching did not cause a significant reduction in CMJ jumping performance [20]. The altered Achilles tendon properties, due to PNF stretching, are likely to have no significant effects on muscle-tendon interaction during the slow stretch-shortening cycle [21]. This is a possible mechanism leading to an unaffected CMJ final height [21].

Similar to a previous report [22], our findings demonstrated that Fz during CMJ increased gradually in time after PNF. Despite PNFlong not changing Fz after the stretching stimulus, PNFshort significantly increased Fz 15 minutes after the stretch (compared to PNFlong) which is in line with a previous report [23], showing that PNF stretching could alter jumping dynamics. The observed increase in Fz after PNFshort could have led to a non-optimal interaction between force production and propulsion time, resulting in no significant change in jumping height. Our results showed that Fz was increased only 15 minutes after the intervention and did not affect jumping performance. Previous findings showed that the isometric contraction included in the PNF procedure decreases tendon stiffness [10]. In contrast, PNFlong, with no significant changes in Fz for most of the jumps and time points, might have caused a more optimal transfer of force to the bone during the stretch-shortening cycle. It is logical to assume that PNFlong led to lower levels of muscle-tendon stiffness, resulting in a non-optimal level of stiffness and an ineffective transfer of force.

Drop jump

Similar to our previous findings [24,25], DJ height did not show any significant changes after both PNF interventions. The DJ height was slightly lower after PNFshort, while PNFlong led to a slightly greater DJ performance at time point 0. These contradictory effects of PNF might reflect the adaptations of the two parts of the plantar flexors. PNFshort leads to greater stretching of the soleus muscle, as opposed to PNFlong, which affects both the soleus and gastrocnemius muscles. This difference implies that the effect of the second condition on the myotendinous system will be greater. As these two synergistic muscle groups act in different patterns during the stretch-shortening cycle, the altered interaction between the main plantar flexors and the tendon might result in different changes in the final performance. The maximum height during DJs represents the optimal interaction between the produced force and its transmission to the bone [26], and a different interaction between muscle and tendon tissues could lead to a submaximal performance. Furthermore, a possible increase in compliance of the plantar flexors after the stretch might reduce the force transmission to the bone and consequently decrease in performance during the stretch-shortening cycle. Normally, a more compliant tendon would restore and reuse a greater quantity of elastic energy during a stretch-shortening cycle, but the time restriction ("as high as fast as") during the DJ might diminish the positive effect of a more compliant tendon.

Another novel finding of this study is that Fz remains almost stable after PNFlong and increases after PNFshort. The increased force production after PNFshort was accompanied by a similar final jumping height, whereas PNFlong did not alter the produced Fz or the final height. Furthermore, a previous study [27] demonstrated that increasing aponeurosis compliance of plantar flexors could alter jumping dynamics due to better use of the force-length. Both the soleus and gastrocnemius muscles, according to their force-length relationship, would shift the force-length relationship to a more optimal length [28], enabling them to produce a greater level of force. It is noteworthy that PNFshort resulted in greater Fz at both time points (0 and 15 minutes) after the stretches, with the jumping height decreasing progressively. It is possible that the interaction between the plantar flexors became non-optimal to control the mechanical coupling of the ankle and the knee joint during the fast stretch-shortening cycle (SSC). Despite the increased storage and force capability due to PNFshort, the neuromuscular system might not have had the time to adapt to these alterations to improve the final performance. After PNFlong, the Fz remains almost the same with small increases in the final jumping height. The unchanged force production of the plantar flexors with the increased storage capacity of the Achilles tendon might result in these effects.

The findings of this study have important practical implications for athletes and coaches involved in sports performance and rehabilitation. Incorporating PNF stretching techniques, either with the knee extended or flexed, can effectively increase ankle ROM, enhancing joint flexibility and potentially benefiting sports performance and injury prevention. While the PNF interventions did not significantly affect vertical jumping, they did influence Fz during certain jumping tasks. None of the Fz alterations translate into improved jumping height. Thus, athletes and coaches seeking to improve explosive performance should carefully consider the type and timing of PNF stretching to optimize jumping performance. Individualizing stretching protocols based on specific sports demands and performance goals can maximize the benefits of PNF stretching and optimize athletic performance, while also considering the potential impact on joint flexibility and muscle-tendon interaction.

Limitations

Despite the fact that the present study included only sixteen male participants, G*Power analysis revealed that this sample size is the minimum required to produce reliable results. Furthermore, the study exclusively focused on male participants; therefore, the results might not be directly applicable to female athletes. Investigating the effects of PNF stretching on females would be beneficial for a more comprehensive analysis. This study primarily focused on measuring ROM, jumping height, and Fz during jumping tests. For a more comprehensive evaluation of the effects of PNF stretching on sports performance, additional outcome measures such as produced force during the PNF, muscle activation, and ultrasound recordings should be included. Finally, investigating the effects of PNF stretching on other muscle groups beyond the triceps surae could provide a more fulfilled understanding of its overall impact on athletic performance.

## Conclusions

To the best of our knowledge, this is the first study comparing the acute effects of isolated stretching of soleus and gastrocnemius muscles at two different lengths to assess their respective effects on jumping ability. The results indicate that PNFshort and PNFlong have the potential to alter the performance of the stretch-shortening cycle due to possible changes in muscle-tendon interaction. The increased Fz during vertical jumps did not significantly affect the final jump height. In conclusion, PNF stretches improved ankle range of motion without any negative effects on jumping performance. These are the primary findings regarding PNF effects in different lengths. Thus, further research is needed to investigate the long-term effects of PNF stretching on athletic performance and the underlying mechanisms that drive these changes.

## References

[1] Guissard N, Duchateau J (2006). Neural aspects of muscle stretching. Exerc Sport Sci Rev.

[2] Nuzzo JL (2020). The case for retiring flexibility as a major component of physical fitness. Sports Med.

[3] Young WB (2007). The use of static stretching in warm-up for training and competition. Int J Sports Physiol Perform.

[4] Chaabene H, Behm DG, Negra Y, Granacher U (2019). Acute effects of static stretching on muscle strength and power: an attempt to clarify previous caveats. Front Physiol.

[5] Trajano GS, Blazevich AJ (2021). Static stretching reduces motoneuron excitability: the potential role of neuromodulation. Exerc Sport Sci Rev.

[6] Blazevich AJ, Gill ND, Kvorning T (2018). No effect of muscle stretching within a full, dynamic warm-up on athletic performance. Med Sci Sports Exerc.

[7] Reid JC, Greene R, Young JD, Hodgson DD, Blazevich AJ, Behm DG (2018). The effects of different durations of static stretching within a comprehensive warm-up on voluntary and evoked contractile properties. Eur J Appl Physiol.

[8] Magnusson SP, Simonsen EB, Aagaard P, Dyhre-Poulsen P, McHugh MP, Kjaer M (1996). Mechanical and physiological responses to stretching with and without preisometric contraction in human skeletal muscle. Arch Phys Med Rehabil.

[9] Wanderley D, Lemos A, Moretti E, Barros MM, Valença MM, de Oliveira DA (2019). Efficacy of proprioceptive neuromuscular facilitation compared to other stretching modalities in range of motion gain in young healthy adults: a systematic review. Physiother Theory Pract.

[10] Kay AD, Husbands-Beasley J, Blazevich AJ (2015). Effects of contract-relax, static stretching, and isometric contractions on muscle-tendon mechanics. Med Sci Sports Exerc.

[11] Reiner M, Tilp M, Guilhem G, Morales-Artacho A, Nakamura M, Konrad A (2021). Effects of a single proprioceptive neuromuscular facilitation stretching exercise with and without post-stretching activation on the muscle function and mechanical properties of the plantar flexor muscles. Front Physiol.

[12] Mahieu NN, Cools A, De Wilde B, Boon M, Witvrouw E (2009). Effect of proprioceptive neuromuscular facilitation stretching on the plantar flexor muscle-tendon tissue properties. Scand J Med Sci Sports.

[13] Konrad A, Gad M, Tilp M (2015). Effect of PNF stretching training on the properties of human muscle and tendon structures. Scand J Med Sci Sports.

[14] Faul F, Erdfelder E, Lang AG, Buchner A (2007). G*Power 3: a flexible statistical power analysis program for the social, behavioral, and biomedical sciences. Behav Res Methods.

[15] Konrad A, Stafilidis S, Tilp M (2017). Effects of acute static, ballistic, and PNF stretching exercise on the muscle and tendon tissue properties. Scand J Med Sci Sports.

[16] Place N, Blum Y, Armand S, Maffiuletti NA, Behm DG (2013). Effects of a short proprioceptive neuromuscular facilitation stretching bout on quadriceps neuromuscular function, flexibility, and vertical jump performance. J Strength Cond Res.

[17] Arampatzis A, Karamanidis K, Morey-Klapsing G, De Monte G, Stafilidis S (2007). Mechanical properties of the triceps surae tendon and aponeurosis in relation to intensity of sport activity. J Biomech.

[18] Fukunaga T, Kawakami Y, Kuno S, Funato K, Fukashiro S (1997). Muscle architecture and function in humans. J Biomech.

[19] Oliveira LP, Vieira LH, Aquino R, Manechini JP, Santiago PR, Puggina EF (2018). Acute effects of active, ballistic, passive, and proprioceptive neuromuscular facilitation stretching on sprint and vertical jump performance in trained young soccer players. J Strength Cond Res.

[20] Christensen BK, Nordstrom BJ (2008). The effects of proprioceptive neuromuscular facilitation and dynamic stretching techniques on vertical jump performance. J Strength Cond Res.

[21] Konrad A, Seiberl W, Tilp M, Holzer D, Paternoster FK (2022). What to stretch? - isolated proprioceptive neuromuscular facilitation stretching of either quadriceps or triceps surae followed by post-stretching activities alters tissue stiffness and jump performance. Sports Biomech.

[22] Kaya F (2018). Positive effects of proprioceptive neuromuscular facilitation stretching on sports performance: a review. J Educ Train Stud.

[23] Pacheco L, Balius R, Aliste L, Pujol M, Pedret C (2011). The acute effects of different stretching exercises on jump perrformance. J Strength Cond Res.

[24] Kendall BJ (2017). The acute effects of static stretching compared to dynamic stretching with and without an active warm up on anaerobic performance. Int J Exerc Sci.

[25] Marrades-Caballero E, Santonja-Medina CS, Sanz-Mengibar JM, Santonja-Medina F (2018). Neurologic music therapy in upper-limb rehabilitation in children with severe bilateral cerebral palsy: a randomized controlled trial. Eur J Phys Rehabil Med.

[26] Maffiuletti NA, Aagaard P, Blazevich AJ, Folland J, Tillin N, Duchateau J (2016). Rate of force development: physiological and methodological considerations. Eur J Appl Physiol.

[27] Kannas TM, Kellis E, Amiridis IG (2012). Incline plyometrics-induced improvement of jumping performance. Eur J Appl Physiol.

[28] Lemos RR, Epstein M, Herzog W (2008). Modeling of skeletal muscle: the influence of tendon and aponeuroses compliance on the force-length relationship. Med Biol Eng Comput.

